# Beta amyloid oligomers and fibrils stimulate differential activation of primary microglia

**DOI:** 10.1186/1742-2094-6-1

**Published:** 2009-01-05

**Authors:** Cindy M Sondag, Gunjan Dhawan, Colin K Combs

**Affiliations:** 1 Department of Pharmacology, Physiology & Therapeutics; University of North Dakota School of Medicine and Health Sciences, 504 Hamline St., Room 118, Grand Forks, ND 58203, USA

## Abstract

**Background:**

Beta amyloid (Aβ) peptides are the major constituents of the senile plaques present in Alzheimer's diseased brain. Pathogenesis has been associated with the aggregated form of the peptide as these fibrils are the conformation readily found in the plaques. However, recent studies have shown that the nonaggregated, soluble assemblies of Aβ have the potential to stimulate neuronal dysfunction and may play a prominent role in the pathogenesis of Alzheimer's disease.

**Methods:**

Soluble, synthetic Aβ1–42 oligomers were prepared producing mainly dimer-trimer conformations as assessed by SDS-PAGE. Similar analysis demonstrated fibril preparations to produce large insoluble aggregates unable to migrate out of the stacking portion of the gels. These peptide preparations were used to stimulate primary murine microglia and cortical neuron cultures. Microglia were analyzed for changes in signaling response and secretory phenotype via Western analysis and ELISA. Viability was examined by quantifying lactate dehydrogenase release from the cultures.

**Results:**

Aβ oligomers and fibrils were used to stimulate microglia for comparison. Both the oligomers and fibrils stimulated proinflammatory activation of primary microglia but the specific conformation of the peptide determined the activation profile. Oligomers stimulated increased levels of active, phosphorylated Lyn and Syk kinase as well as p38 MAP kinase compared to fibrils. Moreover, oligomers stimulated a differential secretory profile for interleukin 6, monocyte chemoattractant protein-1 and keratinocyte chemoattractant when compared to fibrils. Finally, soluble oligomers stimulated death of cultured cortical neurons that was exacerbated by the presence of microglia.

**Conclusion:**

These data suggest that fibrils and oligomers stimulate unique signaling responses in microglia leading to discrete secretory changes and effects on neuron survival. This suggests that inflammation changes during disease may be the consequence of unique peptide-stimulated events and each conformation may represent an individual anti-inflammatory therapeutic target.

## Background

Alzheimer's disease (AD) is a progressive dementia in which one of the defining characteristics is the deposition of extracellular plaques in the brain [1]. While beta-amyloid (Aβ) fibrils, a key component of the neuritic plaques, have been demonstrated to be neurotoxic *in vitro *[2-4], there is a weak correlation between the severity of dementia and plaque load [5-7]. This suggests that something other than fibrillar Aβ is also contributing to the cell loss and dysfunction characteristic of AD. Recently, the focus has shifted somewhat to the soluble form of Aβ, which has also been found in the cortex of AD patients [8-11]. Interestingly, there is a direct correlation between the levels of soluble oligomers isolated from AD brain and the degree of synaptic loss and cognitive impairment [12]. Additionally, Aβ oligomers are demonstrated to be neurotoxic *in vitro *[13-16]. Increasing evidence suggests that neuronal dysfunction in AD may occur prior to the deposition of fibrillar Aβ and it may be mediated by Aβ oligomers [17]. These data are beginning to unravel the contribution of both the oligomers and the fibrils in Aβ-mediated neurotoxicity in AD.

Although some *in vitro *preparations have demonstrated that Aβ peptides can undergo transitions from monomer to oligomer to protofibril *in vitro *[18,19] other studies have indicated that physiologically secreted forms of oligomeric Aβ are much more resistant to extracellular multimerization [20]. Regardless, a number of studies have now demonstrated that oligomeric Aβ is found in varying molecular weight multimers extracellularly in human disease and its rodent models [8,21-23]. The extracellular multimers have been reported to have a plethora of autocrine effects on neurons. Small molecular weight dimer-trimers have been demonstrated to alter LTP formation both *in vitro *and *in vivo *[24-26]. In agreement, other small molecular weight forms ranging from approximately 8–42 kDa, depending upon the study, have demonstrated reversible effects on decreasing LTP, dendritic spine density and length *in vitro *and direct neurotoxicity as well [14,27-29]. These multimers reportedly interact with a specific plasmalemmal protein complex involving activation of the NMDA receptor and subsequent activation of the tyrosine kinase, fyn, to carry-out their detrimental effects [14,30-33]. Larger molecular weight multimers up to 100 kDa have also been reported to have the ability to bind to neurons and decrease neuron viability although the mechanism remains less described [29,34]

In addition to direct neurotoxic effects of Aβ peptides they also have the ability to stimulate glia. In particular, fibrillar Aβ has been demonstrated to further contribute to cell loss via stimulating microglia to release neurotoxic mediators that propagate an inflammatory cycle [35-38]. As with the neuronal toxicity data, there is also accumulating evidence that soluble oligomeric intermediates also mediate a portion of this inflammatory response [39,40]. These data demonstrated microglia are activated differentially by soluble and protofibrillar Aβ compared to fibrillar [40,41]. Moreover, astrocytes are also differentially responsive to the unique peptide conformations [42]. This suggests that a comprehensive study of the effects of nonfibrillar peptide on microglia activation state is warranted analogous to the observations that have now characterized oligomeric neuron stimulation. Microglial activation is a prominent component of AD histopathology and thus it is of significance to understand the various mechanisms through which these cells are stimulated to acquire a reactive phenotype. These data would provide insight into discreet and perhaps early pathophysiology of the AD brain.

In this study we have compared the ability of oligomeric and fibrillar forms of the Aβ peptide to modulate proinflammatory activation of microglia. We compare the *in vitro *microglial response and demonstrate a unique activation profile stimulated by both oligomers and fibrils, including tyrosine kinase activation, mitogen-activated protein kinase (MAPK) activation and secretion of cytokines and chemokines.

## Methods

### Materials

The 4G10 monoclonal anti-phosphotyrosine antibodies were purchased from Millipore (Billerica, MA). Anti-Lyn, anti-Syk, anti-COX-2, anti-phospho-ERK, anti-ERK2 antibody, horseradish peroxidase conjugated secondary antibodies, and protein A/G PLUS-Agarose beads were from Santa Cruz Biotechnology (Santa Cruz, CA). Anti-phospho-p38, anti-p38, anti-phospho-JNK, anti-JNK, and anti-phospho-Lyn were from Cell Signaling (Beverly, MA). Anti-CD68 antibody was from Serotec (Raleigh, NC). KC ELISA, IL-6 ELISA, and MCP-1 ELISA were from R&D Systems (Minneapolis, MN). Anti-Aβ, clone 6E10, was obtained from Covance (Emeryville, CA). Anti-oligomer antibody, A11 was purchased from Invitrogen. (Camarillo, CA). DMEM/F12, Neurobasal media and B27 supplements were purchased from Invitrogen (Rockville, MD). BSA was purchased from Serologicals Corporation (Norcross, GA).

### Preparation of peptides

In order to generate fibrillar and oligomeric peptides for cell stimulation, Aβ1–42 was purchased from Bachem (Torrance, CA) or American Peptide (Sunnyvale, CA). Oligomers were generated as described in Chromy et al [43]. Briefly, Aβ1–42 peptide was dissolved in cold hexafluoro-2-propanol (HFIP) to a final concentration of 1 mM. The peptide was aliquoted and dried under vacuum. The aliquoted peptides were stored at -80degreeC until use. For use in cell experiments, the peptide was dissolved in DMSO to a final concentration of 5 mM then diluted to 100 μM in Ham's/F12 media. The peptide oligomers were then incubated 24 hours, 4 degree C then spun 14,000 rpm, 4 degree C, 10 min. The supernatant was collected as the oligomeric Aβ peptide. Fibrils were prepared by dissolving Aβ1–42 peptides in ddH2O to a final concentration of 250 μM then incubated at 37 degree C for 1 week [44]. Fibrils were resuspended with vigorous trituration prior to removing aliquots for cell stimulation. In order to assess the peptide states under our bioassay conditions, a portion of each preparation was diluted to 20 μM in DMEM/F12 and incubated an additional 48 hours, 37 degree C for subsequent Western analysis. In order to verify that the fibril concentrations employed were accurate following the 1 week fibrillization procedure, five different aliquots of prepared fibril were quantified during different days by Bradford assay to calculate molarities of the solution. This calculated molarity was compared to the predicted molarity based upon the volume of water added to the known mass of purchased peptide used. The difference between predicted molarity and mean calculated molarities from the volume of fibril assayed varied only by 2.4% (predicted 2.5 nM; calculated 2.4 nM ± 0.3). This verified that even though fibrils formed an insoluble precipitate in the solution it was being adequately resuspended for use as a stimulant.

### Tissue culture

Microglia were derived from the brains of postnatal C57BL/6J mice as described previously [45]. Neurons were cultured from cortices of embryonic day 16 (E16) mice (C57BL/6J) as described previously (Sondag and Combs 2006). For co-cultures, neurons were plated at 260 cells/mm^2 ^in 48 well plates. At day 14, media was removed and replaced with Neurobasal media supplemented with B27 components containing microglia (26 cells/mm^2^) and 20 μM Aβ oligomers or fibrils.

Neurons were cultured from cortices of E16 mice (C57B1/6J). Briefly, meninges-free cortices were isolated, trypsinized and plated onto 0.05 mg/mL poly-L-lysine coated tissue culture wells (260 cells/mm^2^) for 14 days *in vitro *before use. Neurons were grown in Neurobasal media with glutamine and B27 supplements (Life Technologies, Rockville, MD) to consistently provide neuronal cultures able to survive for at least one month in vitro. Neuron purity was increased up to 98–99% through a transient treatment with 1 μmol/L AraC during days 1–3. Culture purity is routinely evaluated by cell counting after immunostaining to identify the neuronal cytoskeletal protein, microtubule associated protein 2 (MAP2).

### Cell stimulation

Microglia were stimulated by removing them from growth media into serum-free DMEM/F12 media (2 × 10^6^cells/mL) containing Aβ oligomer or Aβ fibril. Neurons were stimulated by removing growth media and replacing it with serum free media containing Aβ oligomer or Aβ fibril. Cells were stimulated for 5 minutes or 24 hours and total cell lysates were prepared as described below, or cells were stimulated for 24 hours at 37°C and media was collected.

### Non-denaturing electrophoresis

Sample buffer containing no SDS or β-mercaptoethanol was added to Aβ oligomers or Aβ fibrils. Unheated samples were resolved on a 15% polyacrylamide gel in the absence of SDS. Western blotting was performed as described below.

### Immunoprecipitation

To perform immunoprecipitations, cells were lysed in ice-cold radioimmunoprecipitation assay (RIPA) buffer. Lysates were then vortexed, incubated on ice for 15 min, briefly pulse sonicated, and centrifuged (10 min, 4 °C) to remove insoluble material. Primary antibody (1 μg/mg protein) was added to the equal protein amounts from supernatants and incubated 4 h at 4°C. Protein A/G beads (Santa Cruz) were added and incubated an additional 4 hr at 4°C. Beads were washed three times with lysis buffer, and immunoprecipitates were resolved and Western blotted as described below.

### Western blotting

Western blotting of cell or tissue lysate was done as described previously [45]. Briefly, ice cold RIPA buffer was used to lyse cells. Lysates were sonicated and centrifuged (14,000 × g, 4°C, 10 min) to remove insoluble material. Protein concentrations were quantitated and proteins were resolved by SDS-PAGE and transferred to PVDF membranes. Western blots were blocked and incubated in primary antibodies. Blots were washed followed by incubation with HRP-conjugated secondary antibodies. Blots were washed again followed by detection with enhanced chemiluminescence (Pierce, Rockford, IL).

### Enzyme linked-immuno-sorbent assay (ELISA)

Media was collected from microglia following 24 hour stimulation. Levels of mouse interleukin-6, KC, and MCP-1 in the media were determined using commercially available ELISA kits according to the manufacturer's protocol.

### Cell viability assays

#### Lactate dehydrogenase release (LDH) Assay

Media was collected following 24 hour cell stimulation and centrifuged (14,000 × g, 2 min, 25°C). Aliquots were then added to a 96-well plate and LDH concentrations assayed according to manufacturer's instructions (Promega Corporation, Madison, WI). Background absorbance was subtracted from each condition. Conditions were performed with 12 replicates and each experiment was repeated 3 times. Values were averaged (± SD) as a percent of control release.

#### Cell counting assay

As an additional means to assess cell viability, neurons were fixed following 72 h stimulation, stained using a neuron specific anti-MAP2 antibody, and a counting grid was placed under the wells to count stained neuron numbers from four identical fields per condition. MAP2 immunoreactive cells with visible nuclei and processes at least one cell diameter in length, were counted as neurons. The average number of neurons (± SEM) was calculated for each condition. Each experiment was performed in quadruplicate 3 times.

## Results

### Oligomeric and fibrillar Aβ1–42 stimulate a tyrosine kinase-based activation response in microglia

**Figure 1:**
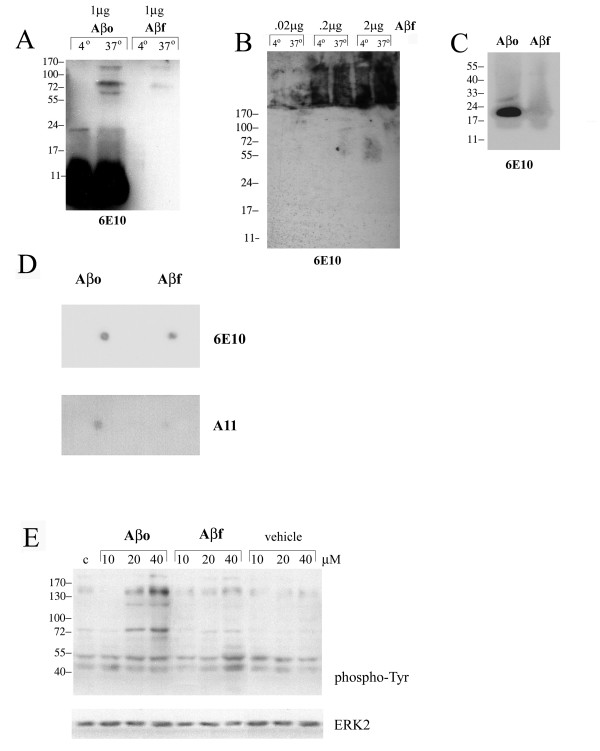
**Oligomeric and fibrillar Aβ stimulated a qualitatively similar and dose-dependent increase in tyrosine phosphorylated protein levels**. Aβ1–42 oligomers (Aβo) and Aβ1–42 fibrils (Aβf) were prepared to a 100 μM concentration via 4 degree incubation overnight (Aβo) or 37 degree incubation 7 days (Aβf) then diluted to 20 μM in DMEM/F12 media and incubated an additional 48 hours at 37 degree C to determine peptide states under bioassay conditions. 1 μg (A, C) or 0.2–2 μg (B) of peptides were separated by (A, B) 15% SDS-PAGE or (C) 15% non-denaturing gel electrophoresis and Western blotted with anti-Aβ antibody, 6E10. (D) Alternatively, prepared Aβo and Aβf were dot blotted (5 μg) each onto PVDF and blotted with anti-Aβ antibody, 6E10, or anti-oligomer antibody, A11. (E) Primary mouse microglia were unstimulated (control) or stimulated with increasing concentrations of Aβo, Aβf, or vehicle. Cells were lysed after 5 min with RIPA buffer. Cell lysates were separated by 7% SDS-PAGE and Western blotted with anti-phosphotyrosine antibody (4G10) and anti-ERK2 antibody (loading control). Antibody binding was visualized by chemiluminescence. Blots are representative of at least three independent experiments.

In order to define the peptides used in our system, we first assessed the state of our oligomer and fibril preparations. To confirm that the Aβ1–42 peptide maintained the appropriate fibrillar or oligomeric conformation throughout the duration of stimulation, we incubated the peptide in media alone at 37 degree C for 48 hours (Fig. 1A). As reported by others, the peptide forms high molecular weight SDS-stable oligomers with incubation but does not adopt a fibrillar conformation [46]. The bulk of the peptide in the oligomeric preparation migrated in the molecular weight range of dimer/trimers (Fig. 1A). As expected, the fibrillar peptide in spite of increasing amounts of loaded protein, did not resolve into the separating gel and remained in the stacking portion of the gel (Fig. 1B). Upon subjecting the peptide to non-denaturing gel electrophoresis (Fig. 1C), we determined again that the fibril was too large to resolve into the separating gel. However, under these conditions the bulk of the oligomers ran as a ~24 kD species rather than the dimer/trimer that was present under the denaturing conditions, suggesting that the dimer/trimers were a result of larger multimer disassembly during the SDS-PAGE. It is likely the 24 kDa form that was observed under the non-denaturing conditions is a more representative formation of the peptide presented to cells during our experimental studies. As further evidence of the oligomeric versus fibrillar properties of our two preparations we performed dot blot Westerns using anti-Aβ antibody, 6E10, versus anti-oligomer antibody, A11 [47], to verify that minimal oligomeric peptide was in our fibrillar preparation (Fig. 1D).

We next compared the ability of fibrillar and oligomeric forms of Aβ1–42 to stimulate primary microglia by treating our cultures with increasing doses of the peptides. Prior work has demonstrated that fibrillar Aβ stimulates a specific tyrosine kinase-based activation response in microglia leading to acquisition of a reactive, neurotoxic phenotype [48-50]. In order to determine whether Aβ oligomers stimulate a comparable response, we first assayed changes in protein phosphotyrosine levels following stimulation. Western blot analysis revealed a dose dependent and qualitatively similar increase in protein phosphotyrosine levels, an indirect measure of tyrosine kinase activity. Interestingly, the oligomers (Aβ_o_) were a qualitatively more potent stimulant than the fibrils (Aβ_f_) (Fig. 1E).

**Figure 2:**
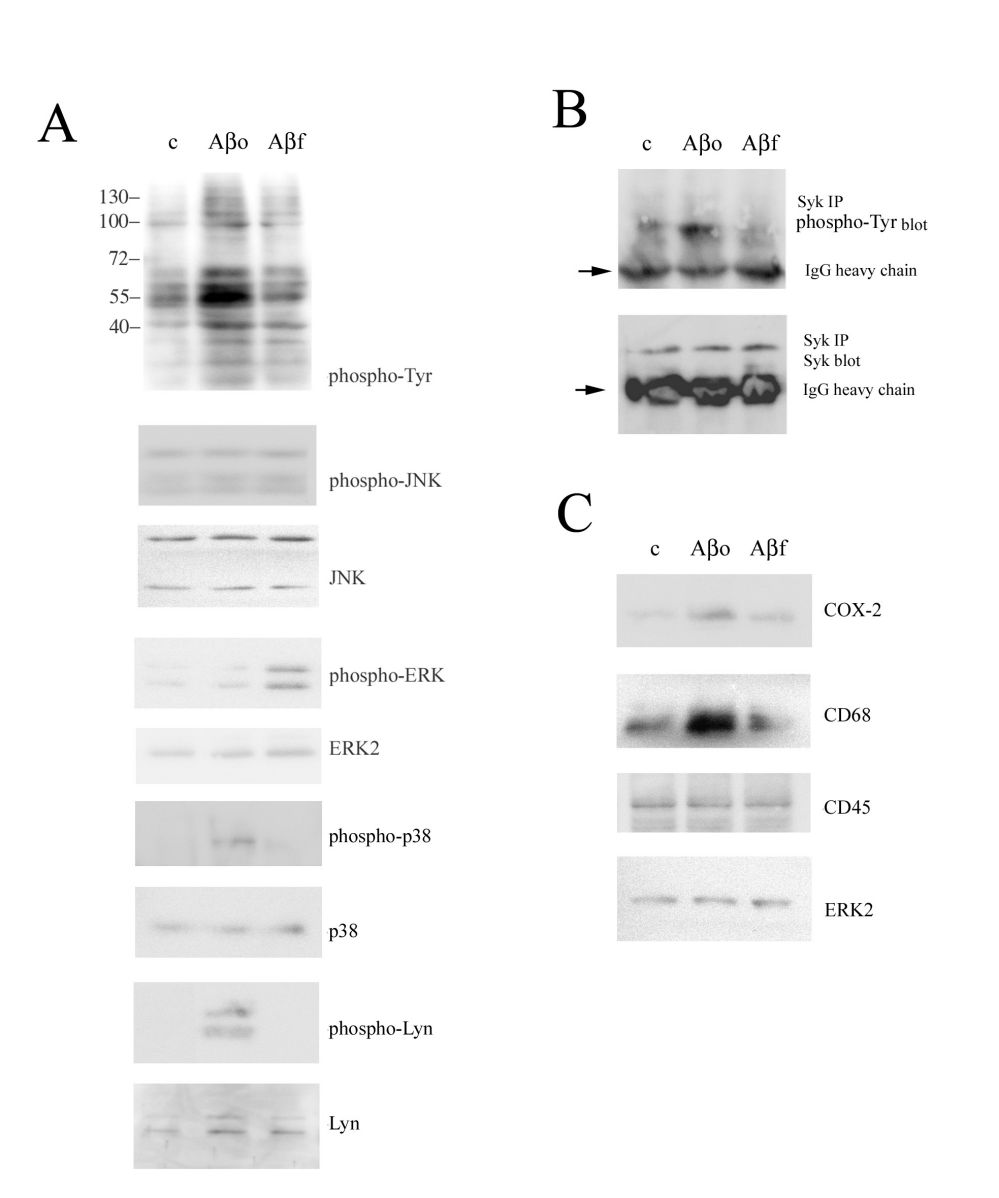
**Aβ1–42 stimulated a conformation-specific MAP and tyrosine kinase signaling response along with increased COX-2 and CD68 protein levels**. Primary mouse microglia were unstimulated (control) or stimulated with 20 μM Aβo or 20 μM Aβf. (A) Cells were lysed after 5 min with RIPA buffer and lysates were separated by 10% SDS-PAGE and Western blotted with anti-phosphotyrosine antibody (4G10), anti-phospho-JNK antibody, anti-JNK antibody (loading control), anti-phospho-ERK antibody, anti-ERK2 antibody (loading control), anti-phospho-p38 antibody, anti-p38 antibody (loading control), anti-phospho-Lyn antibody, or anti-Lyn antibody (loading control). (B) Cells were also lysed after 5 min with RIPA buffer and Syk was immunoprecipitated. Immunoprecipitates were separated by 7% SDS-PAGE and Western blotted with anti-phosphotyrosine antibody (4G10) and anti-Syk antibody. Arrowheads differentiate specific immunoreactivity from IgG heavy chain. (C) Cells were lysed after 24 hrs with RIPA buffer. Cell lysates were separated by 10% SDS-PAGE and Western blotted with anti-COX-2 antibody, anti-CD68 antibody, anti-CD45 antibody and anti-ERK2 antibody (loading control). Antibody binding was visualized by chemiluminescence. Blots are representative of at least three independent experiments.

To continue comparing the activation profile, we tested whether Aβ-mediated stimulation of tyrosine kinases led to subsequent activation of MAPKs since we, as well as others, have demonstrated that Aβ_f _also stimulate increased MAP kinase activities in microglia [49,51-53]. Aβ_f _stimulated increased levels of the active, phosphorylated form of extracellular signal-regulated kinase (ERK) but not p38 or c-Jun N-terminal kinase (JNK) (Fig. 2A). In contrast, Aβ_o _stimulated increased activity of p38 MAPK (Fig. 2A). To further characterize the signaling response associated with Aβ stimulation, we sought to identify proteins that were tyrosine phosphorylated upon stimulation. Previous studies demonstrated the Src family, non-receptor tyrosine kinases Lyn and Syk regulate MAPK activation during Aβ stimulation of monocytes [49]. Therefore, we assessed activation of these specific tyrosine kinases via assaying their phosphorylation state upon stimulation. As expected, levels of the phosphorylated form of both Lyn and Syk increased upon Aβ stimulation, however the activation was specific to the oligomeric form of the peptide (Fig. 2A, B). Collectively, these data demonstrate that the different peptide conformations stimulate similar but distinct signaling responses in microglia.

### Oligomeric and fibrillar Aβ1–42 stimulate unique reactive phenotypes in microglia

**Figure 3:**
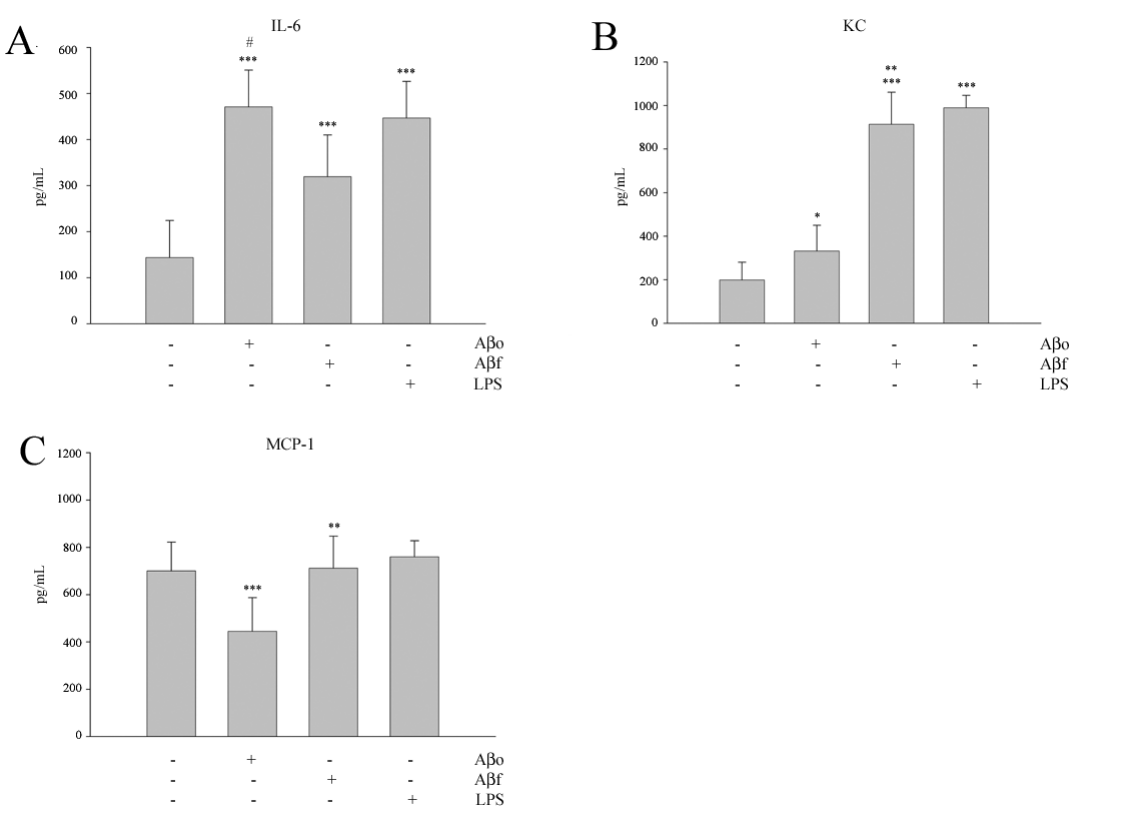
**Aβ1–42 stimulated a conformation-specific increase in proinflammatory cytokine and chemokine secretion**. Primary microglia were unstimulated (control) or stimulated for 24 hours with 20 μM Aβo, 20 μM Aβf, or 25 ng/mL lipopolysaccharide (LPS, positive control). (A) Media were collected and used to perform a preliminary screen of a mouse inflammation antibody array to assess whether relative differences in secretion occurred. In order to quantitate select changes, media was collected and analyzed by (A) mouse IL-6 ELISA, (B) mouse KC ELISA, or (C) mouse MCP-1 ELISA. Data were analyzed by unpaired ANOVA with Tukey's post-test comparison and are expressed as mean +/- SD. Values are representative of three independent experiments (* = p < 0.05 over control, *** = p < 0.001 from control, ** = p < 0.001 from oligomer, # = p < 0.001 from fibril).

Based upon the differences observed in the stimulated signaling response we predicted that the resultant, reactive phenotypes would also be unique. An abundance of literature has described the reactive, secretory microglial phenotype stimulated by Aβ_f _while little is known regarding Aβ_o _effects on these cells [39,40]. Our signaling results above suggested that the resultant phenotypes would differ between Aβ_o _and Aβ_f _stimulations. We first compared protein levels of two typical reactive microglial marker proteins, cyclooxygenase-2 (COX-2) and CD68. In addition, we assessed protein levels of CD45, a tyrosine phosphatase demonstrating increased microglial-like immunoreactivity in AD brains [54]. Oligomeric but not fibrillar Aβ stimulated increased protein levels of both COX-2 and CD68 following 24 hr stimulation, while neither form of the peptide stimulated a change in CD45 levels (Fig. 2C). Based upon this difference, we predicted that the two peptide conformations would stimulate distinct secretory profiles. Using results from a preliminary cytokine/chemokine array profile (Fig. 3A) we elected to quantify secretion of interleukin-6 (IL-6), keratinocyte chemoattractant chemokine (KC, mouse homolog to IL-8), and monocyte chemoattractant protein-1 (MCP-1) following stimulation. Both Aβ_o _and Aβ_f _stimulated increased IL-6 and KC secretion. However, Aβ_o _stimulated a significantly greater amount of IL-6 (Fig. 3A) while Aβ_f _stimulated a significantly greater amount of KC (Fig 3B). Interestingly, Aβ_o _stimulated a decrease in MCP-1 secretion while Aβ_f _had no effect (Fig. 3C). In agreement with the differences observed in stimulated signaling response, these data confirm that the resultant reactive, secretory phenotypes stimulated by Aβ_o _or Aβ_f _are distinct.

### Oligomeric and fibrillar Aβ1–42 stimulations have different neurotoxic effects

**Figure 4:**
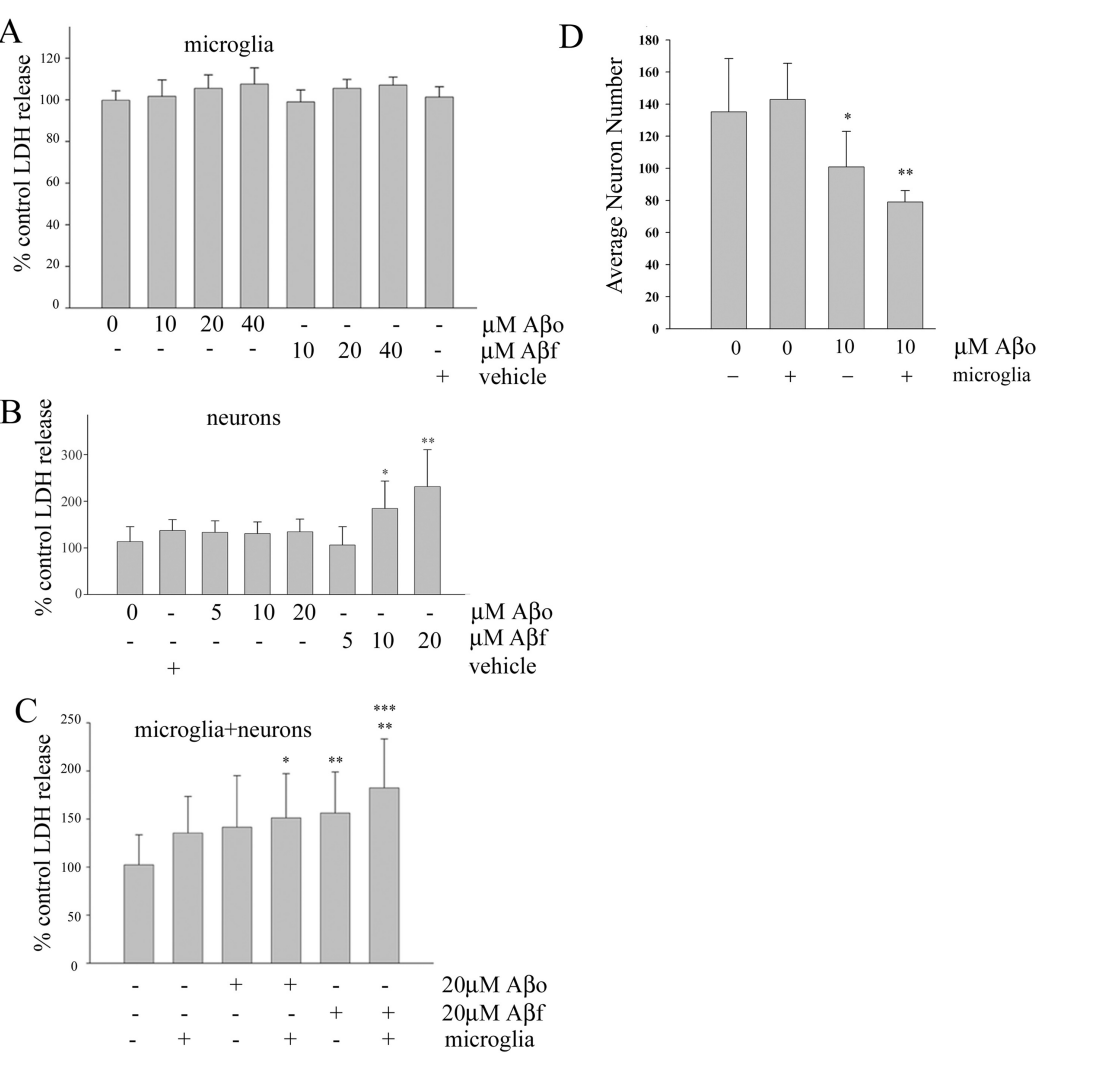
**Aβ1–42 oligomers were only neurotoxic in neuron-microglia co-cultures**. **(**A) Primary microglia, (B) 14 day *in vitro *primary cortical neuron, or (C, D) primary neuron-microglia co-cultures were unstimulated (control) or stimulated for 24 (A-C) or 48 (D) hours with increasing doses of Aβo, Aβf, or vehicle. Media were collected and analyzed by (A-C) LDH release assay to assess viability. Data were analyzed by unpaired ANOVA with Tukey's post-test comparison and are expressed as mean +/- SD. Values are representative of three independent experiments (* = p < 0.05 from control, ** = p < 0.001 from control, *** = p < 0.01 from Aβf+neurons). (D) Alternatively, neurons were fixed after stimulation and immunostained for MAP2 expression and counted to assess viability. Data were analyzed by unpaired ANOVA with Tukey's post-test comparison, expressed as mean +/- SEM, and representative of three independent experiments. (* = p < 0.01 from microglia+neurons, ** = p < 0.001 from microglia+neurons).

We next compared whether the two peptide conformations stimulated microglia to acquire a neurotoxic phenotype. Several prior reports have demonstrated that Aβ_f _stimulate microglia to become neurotoxic [36,49,55] while this remains unclear for Aβ_o_. Using our working concentrations responsible for the stimulatory effects described above we first verified that neither Aβ_o _nor Aβ_f _were toxic to microglia (Fig. 4A). We then tested the direct toxicity of the oligomers and fibrils to primary cortical neuron cultures as there have been conflicting reports about the toxicity of the oligomers *in vitro *[43,56]. After quantitating released LDH into the media it appeared that the oligomers did not stimulate neuronal toxicity under our assay conditions. However, the same assay demonstrated significant fibril dependent toxicity in a dose-dependent manner, as expected (Fig. 4B). Because we have identified several proinflammatory molecules that are secreted by microglia in response to Aβ1–42 stimulation, we tested whether the different conformations of the peptide stimulated microglia to secrete neurotoxic species when they were co-cultured with neurons. As expected, the fibrils stimulated microglia to increase neuron death during a 24 hour stimulation in mixed cultures above that induced by direct neuronal toxicity of the peptide alone (Fig. 4C). Although the oligomers were not toxic alone, as determined by LDH release quantitation, they did stimulate neuronal death in the presence of microglia (Fig. 4C). However, microglia can basally secrete LDH that could act as a confound in our LDH quantitation data and LDH release may not accurately identify cells dying by means other than necrotic death. Therefore, the ability of oligomeric Aβ to kill neurons independent and in the presence of microglia was again examined using a cell counting measure to determine viability. In addition, a longer time period of stimulation, 48 hours, was employed. These data demonstrated that oligomeric peptide alone was toxic to neurons at this longer time period and stimulated a greater degree of cell death when microglia were co-cultured with the neurons (Fig. 4D). This suggests that the longer time period of stimulation or the different assay of viability are required to accurately determine Aβo effects on neurons in our cultures. Collectively, these data demonstrated that the two peptide conformations stimulate distinct effects on neuron viability both directly and indirectly through microglial activation.

## Discussion

There is increasing evidence that Aβ oligomers have an early role in AD pathology before the appearance of amyloid deposits [17]. Importantly, oligomers have been demonstrated to potentiate synaptic loss and inhibit hippocampal long-term potentiation (LTP) *in vitro *and *in vivo *in the absence of any fibril formation [24,57]. Furthermore, transgenic mice overexpressing APP have substantial presynaptic loss before the appearance of Aβ deposits [58,59]. Thus, amyloid fibril formation may be an end stage event in a disease process that is mediated by the effects of the oligomers. To date, there are few reports as to how microglia react to Aβ oligomers and how that compares with fibril-mediated activation [40]. While it is known that Aβ fibrils stimulate microglial activation *in vitro*, it is unclear where this fits in the neuroinflammatory timeline of events in AD. Here we examined the effect of oligomeric and fibrillar Aβ1–42 on proinflammatory activation of microglia *in vitro*. Microglial cultures treated with oligomeric Aβ exhibited different profiles for changes in tyrosine phosphorylated proteins, MAPK activation, and subsequent cytokine and chemokine production than fibril-treated cultures. These results suggest that oligomeric and fibrillar Aβ1–42 may play distinct roles in the proinflammatory activation of microglia demonstrated in AD. While both conformations of the peptide stimulated increased levels of tyrosine phosphorylated proteins, they did so at qualitatively different levels and the resultant phenotypes differed between the two stimuli. We demonstrated that oligomers and fibrils activate the microglia through unique signaling pathways that include activation of specific MAPKs for each form of the peptide. In addition, the propagation of this signaling response through subsequent activation of Lyn and Syk tyrosine kinases is specific to the oligomeric peptide. Again, this reinforces the differences between the ability of the fibrils and oligomers to stimulate microglia and provide potential therapeutic targets to alleviate inflammation associated with AD. Although the peptide comparisons were based upon molarity calculations, it is possible that the surface area of peptide in the two different states is a variable influencing ability to stimulate microglia. For example, the aggregated, insoluble nature of the fibril may diminish the surface area for stimulating microglia in spite of a comparable or even higher molarity comparison to oligomers. With this caveat in mind, fibrils did induce direct, significant neurotoxicity, assayed by LDH release, at concentrations that were inadequate for oligomers (Fig. 4B). This differential neuron effect at similar concentrations, together with the ELISA and signaling data supports the notion that similar concentration comparisons with microglia are reasonable, at least as a starting point, for oligomers versus fibrils.

It is well-documented that Aβ1–42 stimulates increased cytokine secretion from microglia and cytokine upregulation is a key feature demonstrated in AD [60,61]. IL-6 expression is largely increased in AD brain and is believed to have a prominent role in the inflammatory cycle associated with the disease [62]. Interestingly, we show that both Aβ_o _and Aβ_f_stimulate increased IL-6 release from microglia but the soluble assemblies stimulate significantly more than the fibrils. In contrast, the fibrillar conformation stimulates a significantly greater amount of the proinflammatory chemokine, KC, than the oligomer. KC is the murine homolog to IL-8, a chemokine with demonstrated proinflammatory effects that is also upregulated in AD brain [63,64]. These data demonstrate that Aβ1–42 is indeed a stimulus for increased cytokine production in microglia but more importantly it is the specific conformation of the peptide that dictates the nature of the release. The decrease in MCP-1 release upon oligomer stimulation, while somewhat unexpected, is yet another example of how the oligomers and fibrils differ in the means by which they stimulate microglia. It is possible that these data will provide relevant information regarding when in the disease process some of the inflammatory mediators are released. For example, it has been suggested that IL-6 upregulation occurs relatively early in AD, prior to neuritic changes [17]. This is supported by our data which demonstrates the oligomer is a potent stimulus for microglial IL-6 release relative to the fibrils.

We and others have demonstrated that Aβ1–42 is neurotoxic *in vitro *[2-4]. However, there are still conflicting reports as to whether it is the oligomers or fibrils that are the more potent neurotoxin. It is likely that the different assay parameters are affecting the clarity of this outcome. For example, our results demonstrated that oligomeric peptide was toxic to neurons when viability was assessed via cell counting but was not toxic when cell death was determined by measuring levels of LDH released into the media. In addition, primary neuronal cultures and cell lines appear to respond differently to stimulation with Aβ1–42 [56,65]. Also, there may be some discrepancies that can be attributed to the use of the synthetic Aβ1–42 and the consistency of its preparation. We have characterized by denaturing electrophoresis our oligomeric preparation to be approximately dimer-trimer forms initially and multimerizing to several larger molecular weight species with *in vitro *incubation. It is important to point out that our species of Aβ may not be the only disease relevant multimers. For example, recent work by Lesne et al has demonstrated that memory deficits in a transgenic AD mouse model can be produced by a 56 kDa multimer of Aβ [22]. Reports by others have also demonstrated that varying molecular weight oligomers, detected by immunostaining, co-localize with neurons in AD brains [34,66] and bind to neuronal membranes *in vitro *to affect changes in gene expression [34]. Others have demonstrated that smaller molecular weight oligomers have distinct effects on neuron dysfunction [24-26]. Although our preparation is consistent with that employed by others [33] we can not be entirely specific regarding the particular species involved in the stimulated cytokine secretion *in vitro *or the toxicity induced during the 48 hour stimulation due to the clear ability of the peptide to multimerize to higher molecular weight species in our hands. Indeed, the fact that large concentrations (10–20 μM) were needed to stimulate signaling responses and cytokine secretion may well suggest that the particular species involved in stimulating microglia or neuron death may be one of the less abundant molecular weight species in our preparation. Future efforts involving chromatographic separation of the different oligomeric forms followed by acute stimulation of microglia may help define any particular receptor interactions as well as determine whether a distinct form is responsible for stimulating the tyrosine kinase-mediated signaling response.

## Conclusion

This study provides novel information regarding the differences between oligomeric and fibrillar Aβ1–42 stimulation of microglia *in vitro*. Importantly, these data demonstrate multiple proinflammatory mediators that are released upon stimulation with either peptide that likely contribute to the neuropathology evident in AD. These data may not only help develop a timeline for proinflammatory mediator release by microglia in AD, but also may provide critical information with respect to therapeutic strategies for the disease. These data emphasize the necessity to compare side-by-side responses of microglia to both oligomeric and fibrillar peptide conformations to gain an accurate impression of the behavior of these cells during aging and disease as well as provide the framework for logical intervention strategies.

## Competing interests

The authors declare that they have no competing interests.

## Authors' contributions

CS performed the majority of experiments and data analysis, and wrote the initial version of the manuscript. GD performed neuron/microglia co-culture stimulations and assessed viability via immunostaining and counting. CC was involved in conceiving the study and coordinating the experiments. He was responsible for editing and revising the manuscript for the final version.

## References

[B1] Selkoe DJ (1996). Amyloid beta-protein and the genetics of Alzheimer's disease. J Biol Chem.

[B2] Pike CJ, Walencewicz AJ, Glabe CG, Cotman CW (1991). Aggregation-related toxicity of synthetic beta-amyloid protein in hippocampal cultures. Eur J Pharmacol.

[B3] Loo DT, Copani A, Pike CJ, Whittemore ER, Walencewicz AJ, Cotman CW (1993). Apoptosis is induced by beta-amyloid in cultured central nervous system neurons. Proc Natl Acad Sci USA.

[B4] Lorenzo A, Yankner BA (1994). Beta-amyloid neurotoxicity requires fibril formation and is inhibited by congo red. Proc Natl Acad Sci USA.

[B5] Terry RD, Peck A, DeTeresa R, Schechter R, Horoupian DS (1981). Some morphometric aspects of the brain in senile dementia of the Alzheimer type. Ann Neurol.

[B6] Braak H, Braak E (1991). Neuropathological stageing of Alzheimer-related changes. Acta Neuropathol (Berl).

[B7] Dickson DW, Crystal HA, Bevona C, Honer W, Vincent I, Davies P (1995). Correlations of synaptic and pathological markers with cognition of the elderly. Neurobiol Aging.

[B8] Masters CL, Simms G, Weinman NA, Multhaup G, McDonald BL, Beyreuther K (1985). Amyloid plaque core protein in Alzheimer disease and Down syndrome. Proc Natl Acad Sci USA.

[B9] Frautschy SA, Horn DL, Sigel JJ, Harris-White ME, Mendoza JJ, Yang F, Saido TC, Cole GM (1998). Protease inhibitor coinfusion with amyloid beta-protein results in enhanced deposition and toxicity in rat brain. J Neurosci.

[B10] Haass C, Steiner H (2001). Protofibrils, the unifying toxic molecule of neurodegenerative disorders?. Nat Neurosci.

[B11] Kirkitadze MD, Bitan G, Teplow DB (2002). Paradigm shifts in Alzheimer's disease and other neurodegenerative disorders: the emerging role of oligomeric assemblies. J Neurosci Res.

[B12] Lue LF, Kuo YM, Roher AE, Brachova L, Shen Y, Sue L, Beach T, Kurth JH, Rydel RE, Rogers J (1999). Soluble amyloid beta peptide concentration as a predictor of synaptic change in Alzheimer's disease. Am J Pathol.

[B13] Oda T, Wals P, Osterburg HH, Johnson SA, Pasinetti GM, Morgan TE, Rozovsky I, Stine WB, Snyder SW, Holzman TF (1995). Clusterin (apoJ) alters the aggregation of amyloid beta-peptide (A beta 1–42) and forms slowly sedimenting A beta complexes that cause oxidative stress. Exp Neurol.

[B14] Lambert MP, Barlow AK, Chromy BA, Edwards C, Freed R, Liosatos M, Morgan TE, Rozovsky I, Trommer B, Viola KL, Wals P, Zhang C, Finch CE, Krafft GA, Klein WL (1998). Diffusible, nonfibrillar ligands derived from Abeta1–42 are potent central nervous system neurotoxins. Proc Natl Acad Sci USA.

[B15] Hartley DM, Walsh DM, Ye CP, Diehl T, Vasquez S, Vassilev PM, Teplow DB, Selkoe DJ (1999). Protofibrillar intermediates of amyloid beta-protein induce acute electrophysiological changes and progressive neurotoxicity in cortical neurons. J Neurosci.

[B16] Walsh DM, Hartley DM, Kusumoto Y, Fezoui Y, Condron MM, Lomakin A, Benedek GB, Selkoe DJ, Teplow DB (1999). Amyloid beta-protein fibrillogenesis. Structure and biological activity of protofibrillar intermediates. J Biol Chem.

[B17] Klein WL, Krafft GA, Finch CE (2001). Targeting small Abeta oligomers: the solution to an Alzheimer's disease conundrum?. Trends Neurosci.

[B18] Klug GM, Losic D, Subasinghe SS, Aguilar MI, Martin LL, Small DH (2003). Beta-amyloid protein oligomers induced by metal ions and acid pH are distinct from those generated by slow spontaneous ageing at neutral pH. Eur J Biochem.

[B19] Mastrangelo IA, Ahmed M, Sato T, Liu W, Wang C, Hough P, Smith SO (2006). High-resolution atomic force microscopy of soluble Abeta42 oligomers. J Mol Biol.

[B20] Walsh DM, Tseng BP, Rydel RE, Podlisny MB, Selkoe DJ (2000). The oligomerization of amyloid beta-protein begins intracellularly in cells derived from human brain. Biochemistry.

[B21] Kawarabayashi T, Younkin LH, Saido TC, Shoji M, Ashe KH, Younkin SG (2001). Age-dependent changes in brain, CSF, and plasma amyloid (beta) protein in the Tg2576 transgenic mouse model of Alzheimer's disease. J Neurosci.

[B22] Lesné S, Koh MT, Kotilinek L, Kayed R, Glabe CG, Yang A, Gallagher M, Ashe KH (2006). A specific amyloid-beta protein assembly in the brain impairs memory. Nature.

[B23] Oddo S, Caccamo A, Tran L, Lambert MP, Glabe CG, Klein WL, LaFerla FM (2006). Temporal profile of amyloid-beta (Abeta) oligomerization in an in vivo model of Alzheimer disease. A link between Abeta and tau pathology. J Biol Chem.

[B24] Walsh DM, Klyubin I, Fadeeva JV, Cullen WK, Anwyl R, Wolfe MS, Rowan MJ, Selkoe DJ (2002). Naturally secreted oligomers of amyloid beta protein potently inhibit hippocampal long-term potentiation in vivo. Nature.

[B25] Townsend M, Shankar GM, Mehta T, Walsh DM, Selkoe DJ (2006). Effects of secreted oligomers of amyloid beta-protein on hippocampal synaptic plasticity: a potent role for trimers. J Physiol.

[B26] Klyubin I, Walsh DM, Lemere CA, Cullen WK, Shankar GM, Betts V, Spooner ET, Jiang L, Anwyl R, Selkoe DJ, Rowan MJ (2005). Amyloid beta protein immunotherapy neutralizes Abeta oligomers that disrupt synaptic plasticity in vivo. Nat Med.

[B27] Shankar GM, Bloodgood BL, Townsend M, Walsh DM, Selkoe DJ, Sabatini BL (2007). Natural oligomers of the Alzheimer amyloid-beta protein induce reversible synapse loss by modulating an NMDA-type glutamate receptor-dependent signaling pathway. J Neurosci.

[B28] Calabrese B, Shaked GM, Tabarean IV, Braga J, Koo EH, Halpain S (2007). Rapid, concurrent alterations in pre- and postsynaptic structure induced by naturally-secreted amyloid-beta protein. Mol Cell Neurosci.

[B29] Deshpande A, Mina E, Glabe C, Busciglio J (2006). Different conformations of amyloid beta induce neurotoxicity by distinct mechanisms in human cortical neurons. J Neurosci.

[B30] Williamson R, Usardi A, Hanger DP, Anderton BH (2008). Membrane-bound beta-amyloid oligomers are recruited into lipid rafts by a fyn-dependent mechanism. FASEB J.

[B31] Roselli F, Tirard M, Lu J, Hutzler P, Lamberti P, Livrea P, Morabito M, Almeida OF (2005). Soluble beta-amyloid1–40 induces NMDA-dependent degradation of postsynaptic density-95 at glutamatergic synapses. J Neurosci.

[B32] Zhao WQ, De Felice FG, Fernandez S, Chen H, Lambert MP, Quon MJ, Krafft GA, Klein WL (2008). Amyloid beta oligomers induce impairment of neuronal insulin receptors. FASEB J.

[B33] De Felice FG, Velasco PT, Lambert MP, Viola K, Fernandez SJ, Ferreira ST, Klein WL (2007). Abeta oligomers induce neuronal oxidative stress through an N-methyl-D-aspartate receptor-dependent mechanism that is blocked by the Alzheimer drug memantine. J Biol Chem.

[B34] Lacor PN, Buniel MC, Chang L, Fernandez SJ, Gong Y, Viola KL, Lambert MP, Velasco PT, Bigio EH, Finch CE, Krafft GA, Klein WL (2004). Synaptic targeting by Alzheimer's-related amyloid beta oligomers. J Neurosci.

[B35] Gitter BD, Cox LM, Rydel RE, May PC (1995). Amyloid beta peptide potentiates cytokine secretion by interleukin-1 beta-activated human astrocytoma cells. Proc Natl Acad Sci USA.

[B36] Meda L, Cassatella MA, Szendrei GI, Otvos L, Baron P, Villalba M, Ferrari D, Rossi F (1995). Activation of microglial cells by beta-amyloid protein and interferon-gamma. Nature.

[B37] Akiyama H, Barger S, Barnum S, Bradt B, Bauer J, Cole GM, Cooper NR, Eikelenboom P, Emmerling M, Fiebich BL, Finch CE, Frautschy S, Griffin WS, Hampel H, Hull M, Landreth G, Lue L, Mrak R, Mackenzie IR, McGeer PL, O'Banion MK, Pachter J, Pasinetti G, Plata-Salaman C, Rogers J, Rydel R, Shen Y, Streit W, Strohmeyer R, Tooyoma I, Van Muiswinkel FL, Veerhuis R, Walker D, Webster S, Wegrzyniak B, Wenk G, Wyss-Coray T (2000). Inflammation and Alzheimer's disease. Neurobiol Aging.

[B38] Combs CK, Karlo JC, Kao SC, Landreth GE (2001). beta-Amyloid stimulation of microglia and monocytes results in TNFalpha-dependent expression of inducible nitric oxide synthase and neuronal apoptosis. J Neurosci.

[B39] Hashioka S, Monji A, Ueda T, Kanba S, Nakanishi H (2005). Amyloid-beta fibril formation is not necessarily required for microglial activation by the peptides. Neurochem Int.

[B40] Lindberg C, Selenica ML, Westlind-Danielsson A, Schultzberg M (2005). Beta-amyloid protein structure determines the nature of cytokine release from rat microglia. J Mol Neurosci.

[B41] Parvathy S, Rajadas J, Ryan H, Vaziri S, Anderson L, Murphy GM Abeta peptide conformation determines uptake and interleukin-1alpha expression by primary microglial cells. Neurobiol Aging.

[B42] White JA, Manelli AM, Holmberg KH, Van Eldik LJ, Ladu MJ (2005). Differential effects of oligomeric and fibrillar amyloid-beta 1–42 on astrocyte-mediated inflammation. Neurobiol Dis.

[B43] Chromy BA, Nowak RJ, Lambert MP, Viola KL, Chang L, Velasco PT, Jones BW, Fernandez SJ, Lacor PN, Horowitz P, Finch CE, Krafft GA, Klein WL (2003). Self-assembly of Abeta(1–42) into globular neurotoxins. Biochemistry.

[B44] Floden AM, Li S, Combs CK (2005). Beta-amyloid-stimulated microglia induce neuron death via synergistic stimulation of tumor necrosis factor alpha and NMDA receptors. J Neurosci.

[B45] Sondag CM, Combs CK (2006). Amyloid precursor protein cross-linking stimulates beta amyloid production and pro-inflammatory cytokine release in monocytic lineage cells. J Neurochem.

[B46] Gong Y, Chang L, Viola KL, Lacor PN, Lambert MP, Finch CE, Krafft GA, Klein WL (2003). Alzheimer's disease-affected brain: presence of oligomeric A beta ligands (ADDLs) suggests a molecular basis for reversible memory loss. Proc Natl Acad Sci USA.

[B47] Kayed R, Head E, Thompson JL, McIntire TM, Milton SC, Cotman CW, Glabe CG (2003). Common structure of soluble amyloid oligomers implies common mechanism of pathogenesis. Science.

[B48] McDonald DR, Brunden KR, Landreth GE (1997). Amyloid fibrils activate tyrosine kinase-dependent signaling and superoxide production in microglia. J Neurosci.

[B49] Combs CK, Johnson DE, Cannady SB, Lehman TM, Landreth GE (1999). Identification of microglial signal transduction pathways mediating a neurotoxic response to amyloidogenic fragments of beta-amyloid and prion proteins. J Neurosci.

[B50] Nakai M, Hojo K, Yagi K, Saito N, Taniguchi T, Terashima A, Kawamata T, Hashimoto T, Maeda K, Gschwendt M, Yamamoto H, Miyamoto E, Tanaka C (1999). Amyloid beta protein (25–35) phosphorylates MARCKS through tyrosine kinase-activated protein kinase C signaling pathway in microglia. J Neurochem.

[B51] Tan J, Town T, Mori T, Wu Y, Saxe M, Crawford F, Mullan M (2000). CD45 opposes beta-amyloid peptide-induced microglial activation via inhibition of p44/42 mitogen-activated protein kinase. J Neurosci.

[B52] Giovannini MG, Scali C, Prosperi C, Bellucci A, Vannucchi MG, Rosi S, Pepeu G, Casamenti F (2002). Beta-amyloid-induced inflammation and cholinergic hypofunction in the rat brain in vivo: involvement of the p38MAPK pathway. Neurobiol Dis.

[B53] Koistinaho M, Koistinaho J (2002). Role of p38 and p44/42 mitogen-activated protein kinases in microglia. Glia.

[B54] Masliah E, Mallory M, Hansen L, Alford M, Albright T, Terry R, Shapiro P, Sundsmo M, Saitoh T (1991). Immunoreactivity of CD45, a protein phosphotyrosine phosphatase, in Alzheimer's disease. Acta Neuropathol (Berl).

[B55] Qin L, Liu Y, Cooper C, Liu B, Wilson B, Hong JS (2002). Microglia enhance beta-amyloid peptide-induced toxicity in cortical and mesencephalic neurons by producing reactive oxygen species. J Neurochem.

[B56] Roher AE, Chaney MO, Kuo YM, Webster SD, Stine WB, Haverkamp LJ, Woods AS, Cotter RJ, Tuohy JM, Krafft GA, Bonnell BS, Emmerling MR (1996). Morphology and toxicity of Abeta-(1–42) dimer derived from neuritic and vascular amyloid deposits of Alzheimer's disease. J Biol Chem.

[B57] Wang Q, Walsh DM, Rowan MJ, Selkoe DJ, Anwyl R (2004). Block of long-term potentiation by naturally secreted and synthetic amyloid beta-peptide in hippocampal slices is mediated via activation of the kinases c-Jun N-terminal kinase, cyclin-dependent kinase 5, and p38 mitogen-activated protein kinase as well as metabotropic glutamate receptor type 5. J Neurosci.

[B58] Hsia AY, Masliah E, McConlogue L, Yu GQ, Tatsuno G, Hu K, Kholodenko D, Malenka RC, Nicoll RA, Mucke L (1999). Plaque-independent disruption of neural circuits in Alzheimer's disease mouse models. Proc Natl Acad Sci USA.

[B59] Mucke L, Masliah E, Yu GQ, Mallory M, Rockenstein EM, Tatsuno G, Hu K, Kholodenko D, Johnson-Wood K, McConlogue L (2000). High-level neuronal expression of abeta 1–42 in wild-type human amyloid protein precursor transgenic mice: synaptotoxicity without plaque formation. J Neurosci.

[B60] McGeer PL, McGeer EG (1995). The inflammatory response system of brain: implications for therapy of Alzheimer and other neurodegenerative diseases. Brain Res Brain Res Rev.

[B61] Murphy GM, Yang L, Cordell B (1998). Macrophage colony-stimulating factor augments beta-amyloid-induced interleukin-1, interleukin-6, and nitric oxide production by microglial cells. J Biol Chem.

[B62] Griffin WS, Sheng JG, Royston MC, Gentleman SM, McKenzie JE, Graham DI, Roberts GW, Mrak RE (1998). Glial-neuronal interactions in Alzheimer's disease: the potential role of a 'cytokine cycle' in disease progression. Brain Pathol.

[B63] Harada A, Sekido N, Akahoshi T, Wada T, Mukaida N, Matsushima K (1994). Essential involvement of interleukin-8 (IL-8) in acute inflammation. J Leukoc Biol.

[B64] Galimberti D, Schoonenboom N, Scarpini E, Scheltens P (2003). Chemokines in serum and cerebrospinal fluid of Alzheimer's disease patients. Ann Neurol.

[B65] Dahlgren KN, Manelli AM, Stine WB, Baker LK, Krafft GA, LaDu MJ (2006). Oligomeric and fibrillar species of amyloid-beta peptides differentially affect neuronal viability. J Biol Chem.

[B66] Kokubo H, Kayed R, Glabe CG, Yamaguchi H (2005). Soluble Abeta oligomers ultrastructurally localize to cell processes and might be related to synaptic dysfunction in Alzheimer's disease brain. Brain Res.

